# A holistic view on plant effector-triggered immunity presented as an iceberg model

**DOI:** 10.1007/s00018-020-03515-w

**Published:** 2020-04-10

**Authors:** Hans Thordal-Christensen

**Affiliations:** Department of Plant and Environmental Sciences, Copenhagen Plant Science Centre, University of Copenhagen, 1871 Frederiksberg C, Denmark

**Keywords:** Plant immunity, Pathogen effectors, Nucleotide-binding leucine-rich repeat receptors, Lesion mimic mutants, Susceptibility

## Abstract

The immune system of plants is highly complex. It involves pattern-triggered immunity (PTI), which is signaled and manifested through branched multi-step pathways. To counteract this, pathogen effectors target and inhibit individual PTI steps. This in turn can cause specific plant cytosolic nucleotide-binding leucine-rich repeat (NLR) receptors to activate effector-triggered immunity (ETI). Plants and pathogens have many genes encoding NLRs and effectors, respectively. Yet, only a few segregate genetically as resistance (R) genes and avirulence (Avr) effector genes in wild-type populations. In an attempt to explain this contradiction, a model is proposed where far most of the NLRs, the effectors and the effector targets keep one another in a silent state. In this so-called “iceberg model”, a few NLR-effector combinations are genetically visible above the surface, while the vast majority is hidden below. Besides, addressing the existence of many NLRs and effectors, the model also helps to explain why individual downregulation of many effectors causes reduced virulence and why many lesion-mimic mutants are found. Finally, the iceberg model accommodates genuine plant susceptibility factors as potential effector targets.

## Introduction

Crop plants suffer greatly from attack by pathogens, despite the use of a number of control measures. A recent interview-based survey estimated that the global wheat production is reduced by 18% due to fungal pathogens, even though fungicides are commonly applied [1], underlining the significance of plant diseases. NLR-type *R*-genes are broadly used to combat diseases, but they are generally overcome by pathogens within a few years after deployment, while there are only few examples of durable R-genes [2]. Meanwhile, a tremendous amount of highly complex data has been generated over the last decades in this field of molecular plant–pathogen interaction, and it would be beneficial to have a simplified model to accommodate this complexity to help exploiting our insight for designing improved disease resistance.

Genomic studies have added to the complexity by showing that plants have hundreds of genes for intracellular NLR immunity receptors, where for instance hexaploid wheat has 1400 such genes [3, 4]. Although the populations of these genes are very dynamic, the high numbers are maintained in the plant genomes, which indicates that they are active and required. Similarly, searches of genome sequences of filamentous plant pathogens for genes encoding unique and soluble proteins destined for secretion by help of signal peptides have suggested that these pathogens often express several hundred effector proteins [5–8]. Studies of barley powdery mildew effectors have suggested that approximately 20 out of 80 tested contribute significantly to virulence. One-by-one silencing of these 20, in each case reduces virulence by at least 25% [9–13]. What do all these effectors do, and why are the encoding genes maintained in the genomes? If keeping in mind that the genome of this fungus encodes up to 800 effectors [14], it becomes puzzling why these effectors are not redundant [15]. Plant immunity functions in distinct pathways, and targeting of these by more effectors should be influenced by redundancy that prevents one-by-one silencing of these effectors to show major contribution to virulence. In addition, evidence suggests that parallel hormone signaling pathways buffer each other, making the immune system less sensitive to suppression by effectors [16] (see also below). However, immunity can be broken down in consecutive steps, each of which may be able to arrest the pathogen. This can indeed make the role of effectors, dedicated to target such steps, more visible.

Yet, it appears that a better model is required for explaining the existence of the high numbers of effectors. An attempt to do this, while also addressing the high numbers of NLRs, requires that aspects of plant immunity are reviewed. Many NLRs monitor effector targets, and the fact that this does not activate efficient immunity when a virulent pathogen attacks is discussed, as this calls for a set of effectors that suppress this monitoring. Other aspects that contribute to the model is the phenomenon of lesion-mimic mutants, and the striking absence on knowledge on genuine susceptibility factors, which as well may interact with effectors and NLRs. These perspectives are attempted unified in the *iceberg model*.

## The zig-zag model

Molecular interactions between plants and pathogens function as networks of multiple elements, many of which function according to the so-called zig-zag model [17]. Following this model, conserved pathogen molecular patterns are detected by plant plasma membrane-localized pattern-recognizing receptors (PRRs), which activates pattern-triggered immunity (PTI). Well-known PRRs are the Arabidopsis FLS2 and EFR receptors that are activated upon binding of bacterial flagellin and elongation factor EF-Tu, respectively. Both these receptors hetero-dimerize with the co-receptor, BAK1. During the attack by fungal pathogens, chitin oligomers released from the fungal cell wall activate the CERK1–LYK5 receptor kinase complex. Signaling downstream of PRRs involves Ca^2+^ influx, a MAP-kinase cascade and activation of extracellular ROS production by an NADPH oxidase [18]. Meanwhile, pathogens translocate effector proteins and molecules to the plant. Effectors of pathogens, that are adapted to the attacked plant, efficiently interferes with PTI in what will be referred to as 1st level effector triggered susceptibility (1st ETS). Effectors can be extracellular and prevent pattern recognition by PRRs [19, 20], or they can be translocated into the plant cell to target and manipulate immunity proteins, such as the PRRs themselves or immunity signaling components [21]. Now plants have evolved a second class of receptors to monitor whether the pathogen introduces intracellular effectors into the plant cell. These are the intracellular NLR receptors that are activated either by direct binding of effectors or when effectors manipulate immunity proteins, which the NLRs monitor [22]. This 2nd level immunity is referred to as effector-triggered immunity (ETI). To overcome ETI, the pathogen can either eliminate or change the recognized effector, or evolve another effector that suppress this specific monitoring. This latter case will be referred to as 2nd level effector triggered susceptibility (2nd ETS). PTI and ETI involve dramatic transcriptional reprogramming in the plant, including upregulation of defence genes encoding antimicrobial proteins and enzymes for biosynthesis of anti-microbial secondary metabolites, as well as genes encoding proteins associated with signaling hormones (see below). Importantly, ETI generally includes a programmed cell death response, named the hypersensitive reaction (HR) [17, 23].

The zig-zag model has proven very useful for describing the monitoring of PTI effector targets by single NLR-type resistance (R) proteins (see below). However, it does not take into account how the remaining NLRs are prevented from triggering immunity despite they also monitor proteins that are being impacted by effectors during attack from an adapted pathogen. Along the same lines, it fails to address why large numbers of genes for effectors and NLRs are maintained in the genomes, as described above. Using the zig-zag model as basis, the iceberg model addresses these questions.

## NLR function

When single plant genes can be genetically defined to provide protection towards one or more genotypes of a pathogen, they are referred to as resistance (*R*)-genes. *R*-genes most commonly encode NLRs, and based on the zig-zag model, resistance occurs when the NLR-protein detects an effector, either directly or indirectly, and subsequently triggers efficient ETI. It can be speculated whether all *NLR*-genes in a plant species can be genetically revealed as *R*-genes. This is a difficult question, but they should all contribute to resistance to at least one potential invader for them to be maintained in a plant population. This may for instance also be towards nonadapted pathogens (see also below).

A typical NLR has a nucleotide-binding, Apaf1, resistance, CED4 (NB-ARC) central domain, and a leucine-rich repeat (LRR) C-terminal domain. Three major classes of NLRs exist based on their N-terminal domains. These are toll-like, Interleukin-1 receptor domain TIR-NLRs (TNLs), coiled-coil domain CC-NLRs (CNLs), and Resistance to Powdery Mildew 8 (RPW8) CC-type-NLRs (RNLs) [24, 25]. Such typical NLRs are believed to function as molecular switches. In the “off” state, the LRR domain may function as a negative regulator. However, when it binds an effector, or for instance senses a modification of an effector target, a conformational change opens the P-loop of the NB-ARC domain to make an ADP → ATP exchange. This is believed to bring the NLR into its “on” state, where it triggers immunity. Intrinsic ATPase activity of the protein may subsequently hydrolyze the ATP and inactivate the NLR [24, 26]. Some NLRs are translationally fused to additional domains, which are suggested to take over the effector-binding function [3]. Specific amino acid substitutions of the P-loop prevent the ADP–ATP exchange and leave NLRs inactive and dominant negative, while a D → V substitution in the MHD motif of the NB-ARC domain makes NLRs constitutively active [27–32].

A recent structural analysis of the Arabidopsis CNL, ZAR1, has potentially provided a significant advancement of the understanding of how NLRs activate immunity. Wang et al. [33] previously demonstrated that inactive ZAR1 interacts with the pseudokinase RKS1 at the LRR domain. When attacked by *Xanthomonas campestris*, the effector protein AvrAC uridylylates the Arabidopsis PBL2 kinase, which subsequently interacts with RKS1, whereby ZAR1 is activated. Wang et al. [34, 35] recently uncovered that the ZAR1 releases ADP when it associates with RKS1-PBL2^UMP^ on its LRR domain, and they subsequently used cryo-electron microscopy to unravel that dATP or ATP-uptake is required for formation of a pentameric wheel-like supercomplex of the ZAR1–RKS1–PBL2^UMP^ complex. The core of this so-called resistosome is made up of the ZAR1 CC domain, in which the N-terminal ⍺-helix (⍺1) from each ZAR1 molecule protrudes from the wheel-like structure. ⍺1 is required for the ability of the resistosome to induce a cell death response and for its association with the plasma membrane, where the five ⍺1 helices make a tube-like structure believed to form a pore in the membrane, which by unknown mechanisms mediates the cell death response.

ZAR1 is an example of an NLR that appears to mediate immunity without assistance from other NLRs. However, during recent years, it has become apparent that NLRs often function in pairs or networks, where some serve as sensor, and others as helper NLRs. Adachi et al. [36] elegantly reviewed the current insight on this, and list many examples of sensor NLR and how they pair with a shorter list of helper NLRs. No downstream components have been identified for activation of cell death by sensor CNLs, other than helper CNLs and RNLs. This is consistent with the observation that the CNL resistosome complex itself activate cell death. TNLs on the other hand, mediate resistance through the related lipase-like proteins, EDS1, PAD4, and SAG101, where EDS1 complexes with either PAD4 or SAG101 [37, 38]. When Arabidopsis TNL sensors signal through EDS1/PAD4, they use ADR1 (RNL) family members as helpers and primarily mediate transcriptional reprograming, whereas when TNL sensors signal through EDS1/SAG101, they use NRG1 (RNL) family members as helpers, and primarily mediate HR [37–40].

How the signal is transferred from the sensor to the helper NLR is not fully understood. It is speculated that sensor and helper CNLs may enter the same resistosome complex, which alone may activate cell death [36]. In case of TNLs, data indicate that they interact physically with EDS1 lipase-like complexes [41, 42], which in turn appear to interact with at least the NRG1 helper to active HR [38]. However, recently, it has been demonstrated that HR activation by TNLs is dependent of NAD^+^ cleavage and depletion from the cell by NADase activity of the TIR domain, upstream of EDS1 [43, 44].

An example of further branching of NLR networks has been suggested by the finding of an involvement of membrane trafficking components in CNL-mediated immunity. Here the multivesicular body-associated ESCRT-III components, the AMSH3 de-ubiquitinase and the SKD1 AAA–ATPase, were found to be required for resistance and HR mediated by the Arabidopsis CNLs, RPM1 and RPS2, possibly by stabilizing these receptors [45]. AMSH3 was as well required for the lesion-mimic phenotype of *lsd1*, which otherwise is dependent on the helper ADR1 RNLs (see below). Interestingly, the work of Schultz-Larsen et al. [45] suggested that AMSH3 itself is TNL-monitored as knockout of it causes a strong EDS1-dependent lesion-mimic phenotype (see below for lesion-mimic mutants, LMMs).

## Hormones amplify immunity

Both PTI, activated by plasma membrane PRRs, and ETI, activated by NLR-type receptors, are amplified by a hormone signaling network, and involve transcriptional reprogramming activated by MAP kinases in PTI [46, 47] and the EDS1/PAD4 dimer entering the nucleus in TNL-mediated ETI [36]. The activation of transcriptional reprogramming in CNL-mediated ETI is less clear, but can be speculated to be caused by immune signals spreading from cells that have undergone HR. The hormone network involves an interplay of salicylic acid (SA), jasmonic acid (JA), ethylene (Et), and *N*-hydroxypipecolic acid (NHP) [16, 48]. Many enzymes and other proteins, which are regulated during PTI and ETI, have been identified as being important for immunity due to their association with these hormones. In Arabidopsis, immunity-related SA is synthesized in chloroplasts by the shikimate-pathway, in which the isochorismate synthase, SID2, is a well-known enzyme. The MATE transporter, EDS5, is another essential component for the production of immunity-related SA. NHP is synthesized from lysine, which is converted to pipecolic acid by the help of the aminotransferase, ALD1. The flavin-dependent monooxygenase, FMO1, subsequently hydroxylates pipecolic acid into NHP, which is the active signaling hormone. NHP acts synergistically with SA in stimulating immunity [48]. JA is synthesized in the chloroplast and peroxisome from unsaturated fatty acids primarily via 12-oxo-phytodienoic acid. JA is conjugated to isoleucine by JAR1 to make the signaling-active JA-Ile that after translocation to the nucleus stimulates SCF^COI1^-mediated ubiquitination of the transcription repressor, JAZ. Subsequent degradation of JAZ leads to transcription of JA-response genes [49]. Et is synthesized from *S*-adenosyl-methionine in a two-step reaction by ACC-synthase and ACC-oxidase [50]. It is perceived via the Cu^2+^-ions in ETR1 and related proteins, which subsequently release CTR1. This indirectly causes cleavage and transfer of the EIN2 C-terminal to the nucleus, where it interacts with transcription factors to induce Et-response genes [51]. While these hormones integrate in a complex signaling network [52], the sectors controlled by SA, JA, and Et, respectively, are suggested to buffer for one another, making the output immune response less sensitive to blocking of a single sector [16]. Nevertheless, mutants knocked out in individual proteins required for hormone signaling, many of which are mentioned here, have enhanced disease susceptibility [53, 54].

The mechanism by which SA causes immunity-associated transcriptional reprogramming has received significant attention, and the current model is that NPR1 and its paralogues, the redundant NPR3 and NPR4, are master regulators of this [55–58]. These NPRs all have affinity for SA [55, 59], and SA-binding induces cytosolic NPR1 to translocate to the nucleus, where it interacts with TGA transcription factors to induce defense gene expression. NPR1 re-localization to the nucleus involves its nuclear localization signal and that S–S linked oligomers of this protein are reduced to monomers. In the nucleus, differential phosphorylation and sumoylation regulates NPR1’s interaction with the TGA transcription factors [56]. On the other hand, NPR3 and NPR4 interact with TGAs to repress defense gene expression. However, at high SA-level, NPR3 and NPR4-binding of this hormone de-represses gene expression [57].

Interestingly, SA-signaling appears to be self-perpetuating. Treatment of Arabidopsis with SA causes ~ 1500 genes to be upregulated already within the first hour after spraying. By far most of those are regulated by NPR1 and NPR3/4 [57]. Pathogen induction of SA is mediated by induction of the SID2 transcript. Important transcription factors in this process are SARD1 and CBP60g, also required for full activation of many other immunity regulatory genes, including *EDS5* and *NPR1* [60]. Now, the *SARD1* and to a lesser extent also the *CBP60g* transcripts themselves are upregulated by SA in an NPR1- and NPR3/4-dependent manner [57], which indicates that SA signaling is self-perpetuating, since SARD1 the overexpression activates immunity [61]. This self-amplification loop also involves NHP, as the biosynthesis genes *ALD1* and *FMO1* are dependent on SARD1/CBP60g and NPR1/3/4 regulation as well, thereby linking SA and NHP signaling [48, 57, 60, 62, 63]. Among the immunity-related genes upregulated in the loop many encode PRRs and NLRs [57], and in particular overexpression of NLRs can on its own stimulated defense responses, which explains how the self-perpetuating loop amplifies immunity.

## Mechanisms to reduce NLR activation

Plants strike a fine balance between keeping the immune system alert and avoiding damaging effects from its unnecessary activation causing autoimmunity [64–66]. Even nonattacked wild-type plants of *Arabidopsis* may be affected significantly by background activation of the immune system. Tian et al. [67] could show that presence of the CNL, RPM1, had a yield penalty of 9% on seed production in Arabidopsis, which illustrates that it is essential to suppress the NLRs when they are not required for immunity. To this end, NLRs are regulated at several levels: (1) miRNAs and in turn phasiRNAs mediate broad transcriptional and post-transcriptional silencing of *NLR* genes [66, 68]. (2) *NLR* transcript maturation is regulated at the level of intron splicing [64]. (3) Low level of NLR proteins is furthermore obtained by a constitutively active degradation pathway. This is exemplified for the TNL, SNC1, and the CNL, RPS2, where the protein levels in both cases are regulated by the SCF^CPR1^ E3 ubiquitin ligase complex [69, 70] and the MUSE3 polyubiquitin ligase [71]. The loss of SCF^CPR1^ leads to the accumulation of SNC1 and RPS2, and consequently growth retarding autoimmunity. (4) Finally, the activity of NLRs is regulated at the level of (d)ADP-to-(d)ATP exchange and resistosome assembly [26, 34, 35], the latter likely involving the chaperones, SGT1b, RAR1, and HSP90 [72].

## Many lesion-mimic mutants are NLR dependent

For these reasons, it is conceivable that immunity is easily activated by genetic changes, and indeed many autoimmune lesion-mimic mutants (LMMs) have been described. Besides, *cpr1* that has lost the SCF^CPR1^ E3 ubiquitin ligase for SNC1 and RPS2, an amino acid change in SNC1 itself, encoded by *snc1*, interferes with its ubiquitination leading to SNC1 accumulation and lesion-mimic [69]. Furthermore, D → V substitutions in the NB-ARC MHD motif, which makes the NLR constitutively active, also result in lesion-mimic plants. This has been documented for the CNL, RPM1 [30] and the RNL, ADR1-L2 [31].

It is noteworthy that there are LMMs that are caused by NLRs being indirectly activated by modifications or eliminations of the monitored cellular component, which is either an effector target or a component anticipated to be related to effector function. A well-known example is the Arabidopsis *rin4* mutant. RIN4 is an important immunity component and a major target of bacterial effectors. Knockout of RIN4 causes lethality, which is rescued by knockout of the RIN4-monitoring CNL, RPS2, agreeing with the activation of RPS2 by the effector, AvrRpt2, that cleaves RIN4 [73–76]. Other example of LMMs, which are documented to involve an NLR monitoring of an effector-targeted pathway, is the set of mutants, *mekk1*, *mkk1 mkk2*, and *mpk4*. Here all lesion-mimic phenotypes depend on the CNL, SUMM2 [76]. MEKK1, MKK1/2, and MPK4 constitute a MAP kinase cascade that is essential for PTI [77], in which MPK4 is targeted by the *P. syringae* effector, HopAI1. By sensing the phosphorylation status of the downstream substrate, CRCK3, SUMM2 appears to elegantly monitor the whole pathway [78].

On the one hand, a number of LMM have been described where elimination of monitored proteins also activate known NLRs, but effectors have not yet been implicated. Examples are *lsd1* that has an autoimmunity involving the ADR1 RNLs [31, 79], *acd11* dependent on the TNL, LAZ5 [29], and *exo70B1* dependent on TN2, which is a TNL without the LRR domain [80]. Precise primary function of LSD1, ACD11, and EXO70B1 in immunity have not been allocated.

On the other hand, a number of proteins that have well-documented positive functions in Arabidopsis immunity, appears also to be negative regulators of immunity. *PEN* genes are such examples. *PEN1* encodes the syntaxin SYP121, important for preinvasive immunity [81, 82], and *pen1* mutants have a weak autoimmune response [83]. However, when the closely related gene, *SYP122*, is knocked out as well, then strong LMM plants appear [45, 83, 84]. PEN3 encodes an ABC-transporter, also essential for preinvasive immunity [85], and *PMR4* encodes a callose synthase that synthesizes the callose deposited at the site of attack [86–89]. Both PMR4 and PEN3 are negative regulators of immunity, apparent by the lesion mimic phenotypes of *pmr4* and *pen3*. Lastly, AMSH3, required for RPM1 and RPS2-mediated immunity (see above) also appears to be a negative regulator of an NLR, revealed by the fact that *amsh3* knockout plants are LMMs [45, 89]. These lesions, as well as those of *pen1 syp122*, are believed to be caused by TNLs as they in all cases can at least partially be rescued by *eds1* and/or *pad4* mutations [45, 84, 85, 87]. However, none of these monitoring NLRs have been described.

Remarkably, the potential powdery mildew susceptibility component, MLO (see below), may also be NLR-monitored. *mlo* knockout mutants in different species result in lesions in certain genetic backgrounds, and the lesions, but not the powdery mildew resistance of Arabidopsis *mlo2* are PAD4-dependent [90]. This suggests TNL-monitoring.

In summary, many examples of LMMs suggest that genetic modifications of effector targets trigger monitoring NLRs. This triggering may be a direct result from the change or elimination of the target protein as seen for *rin4*, or it may result from the changed activity of the protein as seen for the MAP kinase cascade mutants, where SUMM2 monitors the phosphorylation level of the downstream CRCK3 [78]. The loss of activity is apparently also monitored in the case of the *pen1 syp122* mutant, where high expression of an inactive version of PEN1 does not rescue the lesion-mimic phenotype [83].

The lesion-mimic phenotype of *pen1 syp122* provides an example of a link between immunity and membrane trafficking. Meanwhile, mutation of membrane trafficking-related genes is often detrimental to the plant, which is generally taken as evidence that the encoded protein is vital for development [91]. However, it occurs that these phenotypes are lesion-mimic-like, and indeed the work of Schultz-Larsen et al. [45], showing that the lethality of the knockout of the MVB-associated AMSH3 de-ubiquitinase is partially rescued by *eds1*, provides an example that such assumed development mutants in fact can turn out to be autoimmunity mutants.

## Susceptibility components

Plant susceptibility (S) components are interesting for plant breeding, as they generally cause resistance when knocked out. They come in two flavors: genuine S components that directly serve to promote disease, independently of immunity, and those that indirectly promote disease as they are negative regulators of immunity. The above described mutations in immunity genes, which results in lesion-mimic phenotypes, are of the latter type as loss of them leads to reduced susceptibility. In recent reviews, both types of S components have been described [92–95].

Mutant screens for loss of susceptibility to powdery mildew have been performed successfully [96, 97]. However, it is unclear whether any of the mutated genes encode genuine S components, as the plants generally are LMM-like, indicating negative regulation of immunity. Yet, examples of genuine susceptibility components include the rice SWEET sucrose transporters that are transcriptionally activated by TAL effectors from *Xanthomonas oryzae*. SWEETs directly contribute to the thriving of a pathogen by increasing the extracellular level of sucrose [98, 99]. The amino acid metabolic enzymes, aspartate kinase 2, and dihydrodipicolinate synthase 2 [100], as well as homoserine kinase [101], are also directly required for plant susceptibility to *Hyaloperonospora arabidopsidis*.

Other aspects of susceptibility concern the accommodation of the pathogen in the host tissue, for instance how niches are established for haustoria inside plant cells. Haustoria are surrounded by plant-derived extrahaustorial membranes (EHM), and it has recently been uncovered that the barley powdery mildew EHM has ER-like properties [102] and data suggest that it has a translocon that allows the passage of proteins [103]. This is different from plants attacked by the oomycetes, *H. arabidopsidis* and *Phytophthora infestans*. Here the EHM share features with the plasma membrane and the tonoplast [104, 105]. Yet, no detailed insight is available on how EHMs are generated, nor have any S components been involved in these processes.

Having said this, the ROP GTPase RACB is positively required for the powdery mildew fungus to develop haustoria in barley epidermal cells. RACB is involved in microtubule organization and detailed studies of RACB regulation has documented that this GTPase organizes the cell polarity to allow establishment of the haustorial complex [106, 107]. Furthermore, it is speculated whether the broadly occurring MLO protein is a genuine S component for powdery mildew fungi, potentially involved in establishing the haustorial complex. The absence of MLO confers strong and durable resistance, observed as preinvasive immunity, to powdery mildew. This resistance has been used in barley for decades and has more recently emerged in many other plant species. Homozygous *mlo* mutants often suffer from pleiotropic effects including spontaneous immune responses and early senescence. Here *mlo* alleles conferring stronger resistance generally also confer stronger pleiotropic effects [108]. This has complicated the deciphering of whether the resistance is due to expression of autoimmunity or whether MLO is a genuine S component. However, the fact that lesions, and not the preinvasive immunity, in Arabidopsis *mlo2* are dependent on PAD4 and intact SA signaling [90] strongly advocate for MLO being a genuine S component required for powdery mildew invasion, and that *mlo2* at the same time is an LMM due to monitoring by a TNL. The first barley cultivars with this type of powdery mildew resistance suffered severely from lesion, but breeding efforts have overcome this problem without losing the effective powdery mildew resistance, despite the same *mlo* alleles being used [109]. It is hypothesized that this breeding effort has selected favorable *NLR* alleles encoding receptors responding less to the loss of MLO. This may well have been possible due to the high variation in the plant NLR populations.

These thoughts raise the idea that not only immunity components, but also plant genuine S components are monitored by NLRs. This idea is also stimulated by the rationale that effectors should target S components. In fact, Arabidopsis MLO2 is indeed targeted by the bacterial effector HopZ2 [110]. The primary function of the seven-transmembrane-helix plasma membrane MLO protein is not known, and if the NLR-monitoring hypothesis is correct then MLO appears not to play vital roles for the plant. Barley RACB and the associated proteins that regulate the plant cell cytoskeleton to accommodate the powdery mildew haustorium are other examples of S components [106, 107]. Indeed, RACB is targeted by the fungal effector ROPIP1, which destabilizes the host cell microtubules, possibly to facilitate fungal entry and haustorium formation [111]. It is expected that much more plant developmental proteins will be revealed as S components, which pathogens manipulate by help of effectors to gain nutrients or to establish a niche in the host cell. Such S components are well-known in animals and humans, where for instance membrane trafficking-related proteins are targeted by effectors and exploited by intracellular bacteria for establishing “vacuoles” for them to dwell in and thereby escape the host immune system [112, 113].

## Pathogen effectors

As referred to above, pathogens transmit effectors to the host to manipulate host processes important for the interaction between the organisms. Many excellent reviews describe effectors, the corresponding NLRs, and the primary targets (e.g., [114-117]). Yet, efforts to describe why in particular filamentous pathogens express hundreds of effectors have been sparse. To study this, it would be required not only to analyze those effectors that are recognized by R-proteins, but to take an unprejudiced approach for studying the effectors encoded by the pathogen genomes. Such an approach has been followed intensely in two articles. Mukhtar et al. [118] and Weßling et al. [119] did systematic yeast 2-hybrid searches for Arabidopsis targets of effectors from a bacterial pathogen and from downy and powdery mildew filamentous pathogens, all adapted to this host. Here, hundreds of effectors from these pathogens were used as preys to find long lists of targets, and interestingly many of them were targeted several times and by the evolutionarily very diverse pathogens. Furthermore, only very few effector targets were NLRs, while more than 150 were proteins not previously associated with pathogen interactions. Meanwhile, many of those in turn interacted with NLRs, supporting the notion that effectors often are indirectly monitored. Interestingly, more than a hundred effector targets were tested for their role in the interactions with the three pathogens using knockout mutant plants. About 20% of these mutants had enhanced susceptibility, while another 20% had enhanced resistance [118, 119]. The latter may reflect that these 21 genes encode genuine S components and/or that they encode NLR-monitored effector targets.

## Effectors that perform 2nd ETS

This work by Mukhtar et al. [118] and Weßling et al. [119] suggest that NLR recognition of effectors in most cases is indirect via an effector target. Because knockout of some of these targets may cause NLR-mediated enhanced resistance, it is assumed that effector-binding will also activate these NLRs. Yet the pathogens used in the studies successfully overcome Arabidopsis’ immunity and cause disease. *How would this be possible with several of their effectors activating NLRs,* i.e., *ETI*? The answer is likely to be that these pathogens also express effectors that can suppress ETI, and mediate 2nd ETS. Examples of this have been revealed for a number of R-proteins. *P. syringae* effector AvrRpt2 has 2nd ETS activity against ETI mediated by Arabidopsis, RPM1, since it cleaves RIN4, and thereby it prevents RPM1 from responding to AvrRpm1-induced RIN4 phosphorylation [17, 73, 120]. Similarly, the Avr1 effector from *Fusarium oxysporum* suppresses resistance in tomato mediated by the R-proteins I-2 and I-3, while it activates resistance mediated by I and I-1 [121, 122]. Based on a genetic segregation study of the wheat powdery mildew fungus, another 2nd ETS effector was uncovered. Here, “suppressor of Avr”, SVRPM3^A1/F1^ suppressed distinct resistances mediated five PM3 NLRs encoded by an allelic series of *R*-genes and triggered by distantly related AVRPM3s [8, 123, 124]. *Leptosphaeria maculans* effector AvrLm4-7 suppresses resistance mediated by R-protein, Rlm3, after recognition of AvrLm3 in oil seed rape [125]. Finally, nine out of twenty *Puccinia graminis* and *P. striiformis* wheat rust effectors suppressed HR in *Nicotiana benthamiana*. Here different heterologous NLR/Avr expressions activated HR, which the rust effectors suppressed in specific manners [126]. The fact that effectors exist that mediate 2nd ETS in association with NLRs, that by-the-way are genetically defined as R-proteins, suggests that 2nd ETS effectors, which are not easily revealed genetically, also occur. They should be required for pathogens to suppress ETI, activated when other effectors target and manipulate host proteins that are monitored by NLRs. With the high numbers of effectors, effector targets and NLRs, it is conceivable that many such 2nd ETS effectors may exist. Coming back to the question from the “Introduction” regarding the many effectors that by individual silencing can be documented to have significant contribution to virulence, an important part of the answer may be 2nd ETS. Silencing an effector with such a function can have severe consequences for the pathogen, as it will result in ETI, an effect not hidden by redundancy. This could help explain why some pathogens maintain large numbers of genes encoding effector-like proteins.

It is a question, whether pathogens have “silver bullet” effectors that once and for all stop the immune system. Indeed, this may in broad sense be the case for many necrotrophic pathogens, where no NLR-type *R*-genes have been found [127]. However, even though SVRPM3^A1/F1^ suppresses ETI activated by closely related NLRs, other *R*-genes can confer resistance against wheat powdery mildew isolates expressing SVRPM3^A1/F1^, showing that this is not a silver bullet effector [8, 123, 124]. This and other examples above, in turn indicates that 2nd ETS effectors function at the stage of effector target monitoring. It is as well in agreement with the fact that CNLs, like ZAR1 appear to activate HR themselves, and; therefore, it is difficult to imagine effector targets downstream of CNLs. The current insight into the interplay between sensor and helper NLRs is insufficient to suggest that 2nd ETS effectors interfere with ETI activated via such combined NLR function. For TNLs, the downstream EDS1/SAG101 or EDS1/PAD4 step can be envisioned to be effector targeted. Meanwhile, it was discussed above that hormone signaling amplify ETI, and effectors indeed target these signaling components [117, 128]. However, since hormone signaling only amplify immunity, effector inhibition of hormone signaling should not be detrimental to ETI. In fact, it is likely that pathogen have effectors dedicated to reduce the hormone amplification of ETI in general. The 69 (out of 91 tested) *Puccinia striiformis* f.sp. *tritici* effectors newly described to suppress Bax-induced programmed cell death in *N. benthamiana* are potentially such effectors [129].

## The iceberg model

As discussed, plants have large numbers of sensor NLRs and filamentous pathogens likewise have large numbers of effectors. These effectors have targets in the plant, many of which in turn are NLR-monitored according to the zig-zag model. Effector targets will often be important for immunity, but as suggested above, another set of effector targets will be S components, which pathogens must manipulate to sustain themselves. Both types of targets are likely to be NLR monitored.

**Fig. 1:**
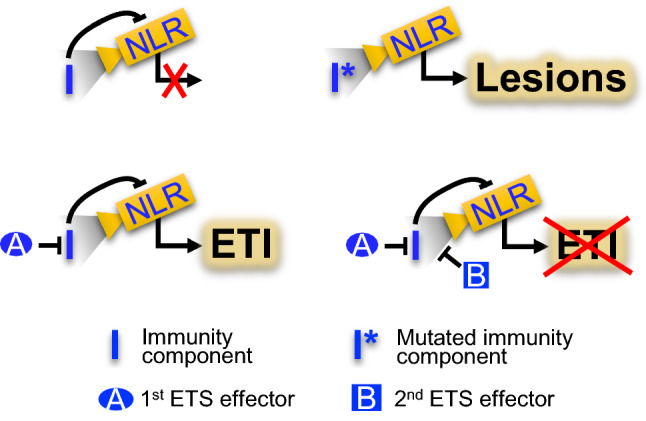
Different stages of a single interaction unit. “NLR” monitors effector target “I”, important for immunity, and activates lesions when “I” is affected by mutations or ETI when “I” is influenced by the 1st ETS effector “A”. Effector “B” prevents activation of NLR, whereby “B” confers 2nd ETS

These proteins function in “interaction units”. In a plant, attacked by a pathogen most interaction units will remain silent. Such silent units in their minimal form consist of an effector target, a monitoring NLR that the effector target suppresses, a 1st ETS effector, and a 2nd ETS effector, where the latter protein causes the silence, as it prevents the NLR from activating immunity (Fig. 1).

**Fig. 2:**
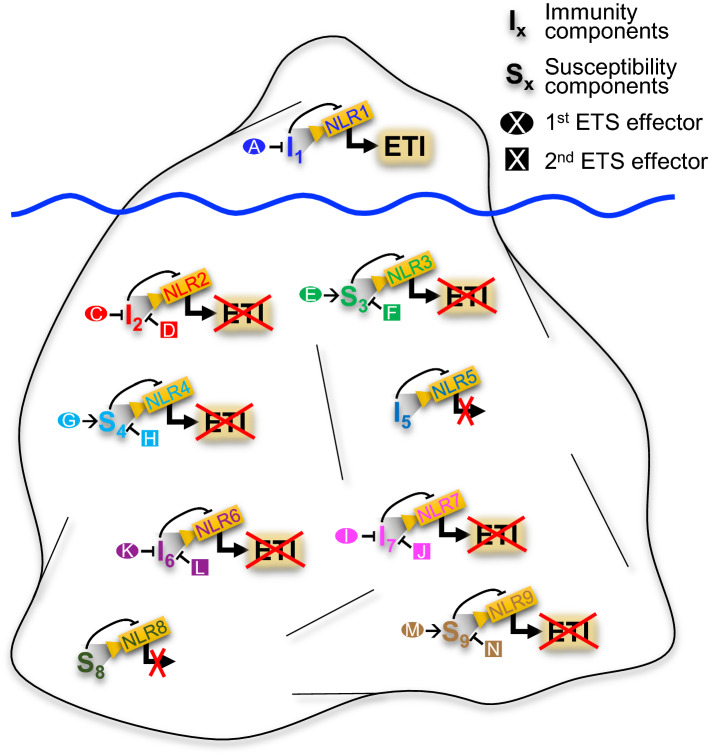
The iceberg model. In plants interacting with, e.g., powdery mildew fungi, many effector target/NLR interaction units are suggested to be silent due to 2nd ETS. Only a few NLR and effector genes segregate genetically as R-genes and Avr-genes, as those encoding NLR1 and effector “A”. Most NLRs are not revealed genetically, since they appear in “silent” interaction units. “I” and “S”, effector targets important for immunity and susceptibility, respectively

In a population of plants interacting with an adapted pathogen, many such silent units will occur. An iceberg model is proposed to visualize that these many silent units do not become apparent in genetic studies of wild-type populations of plant and pathogens. They are pictured to be under the surface in the “bottom” of the iceberg. If a pathogen fails to express a functional 2nd ETS effector, the corresponding interaction unit may be genetically visible through allele variants of the 1st ETS effector gene and the NLR-gene that in this case will be an *R*-gene. This unit will be in the “top” of the iceberg (Fig. 2). The model illustrates how it is possible for many effectors to contribute significantly to virulence as demonstrated by their individual silencing, and how the phenotype of LMMs is dependent on specific NLRs.

Nonadapted pathogens have smaller or larger numbers of effectors that target plant proteins inefficiently. The plant is said to be “nonhost” for these pathogens, towards which it has efficient and durable nonhost resistance. Schulze-Lefert and Panstruga [130] suggested that nonhost resistance in plants, that are remotely related to the pathogen’s host, is based on PTI. The reason should be that the 1st ETS effectors are not able to inhibit the components of this form of immunity. According to this iceberg model, the inefficient effectors can be both 1st and 2nd ETS types, and the nonhost resistance can be a combination of PTI and ETI.

Individual pathogens will not have effectors to target all NLR-monitored immunity and susceptibility components. Therefore, there will be many nontargeted, and thus silent, effector target/NLR sets in the bottom of the iceberg.

As suggested above, a silent interaction unit consists in its minimal form of four components. However, other interaction units are predicted to involved sensor/helper NLRs or higher order complexes of NLRs [36]. Besides, single immunity/susceptibility components may be targeted by more effectors [118, 119]. The iceberg model is considered to be able to accommodate such more complex units, and the future will show whether it can contribute to a holistic view of these, for instance involving predicted effector targeting of recurrent helpers as well as single effectors targeting more immunity/susceptibility components.

Does the iceberg model explain why plants and filamentous pathogens have large numbers of NLRs and effectors? Possibly, yes. The model suggests that pathogens benefit from more 1st ETS effectors, and that plants in the arms race have benefitted from more NLRs, which in turn require more 2nd ETS effectors, stimulating evolution of more NLRs, etc. In this way, plants and pathogens have force one another to undergo gene amplifications leading to the large gene numbers. In other words, in the arms race during the co-evolution of plants and their adapted pathogens, more and more silent interaction units have been placed in the bottom of the iceberg.

## Conclusion

An iceberg model is suggested to unify many aspects of ETI and to provide a holistic view of this level of immunity. From the model, a number of thoughts appear that may affect our understanding of the plant immune system and molecular interactions of plants and pathogens:

- Many effectors can be shown in silencing studies to contribute significantly to virulence. The iceberg model explains the risk of mis-interpreting this to be due to suppression of PTI, as it suggests many such effectors will be 2nd ETS effectors, which contribute indirectly to virulence and not necessarily by affecting the primary activity of the target.
- Knockout mutants affecting cellular processes are often lethal, which is generally taken as evidence that the processes are indispensable. There will be cases where the lethality rather is due to the activation of monitoring NLRs, since the knocked out proteins in fact are pathogen effector targets.
- A sub-group of these lethal mutants may be affected in susceptibility components, which are essential for pathogens. It is potentially possible to exploit these mutations for resistance, provided the corresponding NLR genes are knocked out as well.
- Nonhost resistance is efficient and durable, and often referred to as an ideal protection against disease. This model can help us decipher individual components of nonhost resistance and learn how to exploit these towards adapted pathogens.
- Searching for novel R-genes, by screening collections of plant genotypes for individuals showing lesions after introduction of single effector in the absence of the pathogen, will hardly be a good strategy. The reason is that the pathogen is also likely to express corresponding 2nd ETS effectors, which will suppress the specific NLR-mediated recognition.
- Mutant studies of immunity have favoured revealing of components, which are not NLR-monitored in the used gene background. Examples may be NPR1, EDS5, SID2, ALD1, and FMO1. Potentially, NLR-monitored immunity components are PEN1, PEN3, PMR4, and AMSH3.

## Abbreviations

AAA-ATPase: ATPases Associated with diverse cellular Activities-ATPase
ABC: ATP-binding cassette
ACC: 1-AminoCyclopropane-1-Carboxylic acid
ACD11: Accelerated Cell Death11
ADR1: Activated Disease Resistance
ALD1: AGD2-Like Defense response protein1
AMSH3: Associated Molecule with the SH3 domain of STAM 3
Apaf1: Apoptotic protease activating factor1
Avr: Avirulence
BAK1: Brassinosteroid insensitive1-Associated receptor Kinase1
CBP60g: Calmodulin-Binding Protein 60G
CC: Coiled Coil
CED4: CEll Death abnormality4
CERK1: Chitin Elicitor Receptor Kinase1
CNL: CC-NLR
CPR1: Constitutive expresser of Pathogenesis-Related genes1
CRCK3: Calmodulin-binding Receptor-like cytoplasmic Kinase3
CTR1: Constitutive Triple Response1
EDS1/5: Enhanced Disease Susceptibility1/5
EFR: EF-Tu Receptor
EF-Tu: Elongation factor-Tu
EHM: ExtraHaustorial Membrane
EIN2: Ethylene INsensitive2
ER: Endoplasmic Reticulum
ESCRT: Endosomal Sorting Complexes Required for Transport
Et: Ethylene
ETI: Effector-Triggered Immunity
ETR1: EThylene Response1
ETS: Effector-Triggered Susceptibility
EXO70B1: EXOcyst complex component70B1
FLS2: Flagellin Sensing2
FMO1: Flavin-dependent MonoOxygenase1
HopAI1/Z2: Hrp-dependent Outer ProteinAI1/Z2
HR: Hypersensitive Response
HSP90: Heat Shock Protein 90 kDa
JA-Ile: Jasmonic Acid-Isoleucine
JAR: Jasmonic Acid Resistant
JAZ: Jasmonate Zim domain protein
LAZ5: LAZarus5
LMM: Lesion-Mimic Mutant
LRR: Leucine-Rich Repeat
LSD1: Lesions Simulating Disease1
LYK5: LYsin motif receptor Kinase5
MAP: Mitogen-Activated Protein
MATE: Multidrug And Toxin compound Extrusion transpoter
MEKK1: Mitogen-activated protein kinase Kinase Kinase1
MKK1/2: Mitogen-activated protein Kinase Kinase1/2
MLO/2: Mildew resistance Locus O/2
MPK4: Mitogen-activated Protein Kinase4
MUSE3: MUtant, *snc1*-Enhancing3
MVB: MultiVesicular Body
NAD: Nicotinamid Adenine Dinucleotide
NADPH: Nicotinamid Adenine Dinucleotide Phosphate
NB-ARC: Nucleotide-Binding, Apaf1, Resistance, CED4
NHP: N-Hydroxypipecolic Acid
NLR: Nucleotide-binding Leucine-rich Repeat
NPR1/3/4: Nonexpressor of Pathogenesis Related genes1
NRG1: N Requirement Gene1
PAD4: PhytoAlexin Deficient4
PEN1/3: PENetration1/3
PBL2: PBs1-Like protein2
PMR4: Powdery Mildew Resistance4
PRR: Pattern Recognition Receptor
PTI: Pattern-Triggered Immunity
R: Resistance
RACB: RAt sarcoma-related C botulinum toxin substrateB
RAR1: Required for MlA Resistance
RIN4: RPM1 interacting protein 4
RKS1: Receptor-like Protein Kinase1
RNL: Rpw8-NLR
ROPIP1: ROP Interactive Peptide 1
ROP: RhO in Plants
ROS: Reactive Oxygen Species
RPM1: Resistance to *Pseudomonas syringae* pv. *Maculicola*1
RPS2: Resistance to *Pseudomonas Syringae*2
RPW8: Resistance to Powdery Mildew 8
S: Susceptibility
SA: Salicylic Acid
SARD1: SAR Deficient1
SAG101: Senescence-Associated Carboxylesterase 101
SCF^COI1^: Skp1/Cullin/F-box^COronatin Insensitive1^
SCF^CPR1^: Skp1/Cullin/F-box^Constitutive expresser of Pathogenesis^^−^^Related genes1^
SGT1b: Component of Skp1-Cullin–F-box ubiquitin ligase
SID2: Salicylic acid Induction Deficient2
SKD1: Suppressor of K+ transport growth Defect1
SNC1: Suppressor of *npr1-1*, Constitutive1
SUMM2: SUppressor of *mkk1 mkk2*, 2-1
SVRPM3^A1/F1^: Suppressor of AvrPM3^A1/F1^
SWEET: Sucrose transporter
SYP121/122: SYntaxin in Plant121/122
TAL: Transcription Activator-Like
TGA: TGACG-sequence-specific DNA-binding protein
TIR: Toll-like, Interleukin-1 Receptor
TNL: TIR-NLR
ZAR1: HopZ-Activated Resistance1
